# Supplementary figures and images for: Application of Magnetized Ionized Water and Bacillus subtilis Improved Saline Soil Quality and Cotton Productivity
Source: Plants (Basel). 2024 Sep 2;13(17):2458. doi: 10.3390/plants13172458 (PMC11397375; doi:10.3390/plants13172458)

## Supplementary data

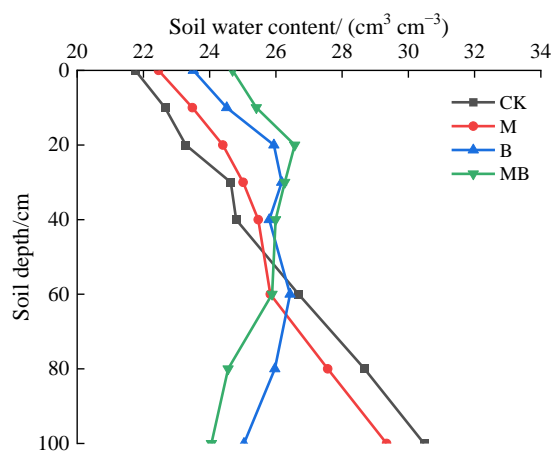

Figure S1. Soil water content during cotton boll stage.

Supplement: Supplementary file 1 [file plants-13-02458-s001.zip › plants-3143860-supplementary.pdf]
